# Lab-based and diagnosis-based chronic kidney disease recognition and staging concordance

**DOI:** 10.1186/s12882-019-1551-3

**Published:** 2019-09-14

**Authors:** Clarissa J. Diamantidis, Sarah L. Hale, Virginia Wang, Valerie A. Smith, Sarah Hudson Scholle, Matthew L. Maciejewski

**Affiliations:** 1 Division of General Internal Medicine, Department of Medicine, Duke University School of Medicine, Durham, USA; 2 Division of Nephrology, Department of Medicine, Duke University School of Medicine, Durham, USA; 3 Department of Biostatistics and Bioinformatics, Duke University School of Medicine, Durham, USA; 4 Department of Population Health Sciences, Duke University School of Medicine, Durham, USA; 5 Durham Center of Innovation to Accelerate Discovery and Practice Transformation (ADAPT), Durham Veterans Affairs Medical Center, Durham, USA; 6 National Committee for Quality Assurance, Washington, DC USA

**Keywords:** Medicare, Beneficiary, Chronic kidney disease, Nephrology

## Abstract

**Background:**

Chronic kidney disease (CKD) is often under-recognized and poorly documented via diagnoses, but the extent of under-recognition is not well understood among Medicare beneficiaries. The current study used claims-based diagnosis and lab data to examine patient factors associated with clinically recognized CKD and CKD stage concordance between claims- and lab-based sources in a cohort of Medicare beneficiaries.

**Methods:**

In a cohort of fee-for-service (FFS) beneficiaries with CKD based on 2011 labs, we examined the proportion with clinically recognized CKD via diagnoses and factors associated with clinical recognition in logistic regression. In the subset of beneficiaries with CKD stage identified from both labs and diagnoses, we examined concordance in CKD stage from both sources, and factors independently associated with CKD stage concordance in logistic regression.

**Results:**

Among the subset of 206,036 beneficiaries with lab-based CKD, only 11.8% (*n* = 24,286) had clinically recognized CKD via diagnoses. Clinical recognition was more likely for beneficiaries who had higher CKD stages, were non-elderly, were Hispanic or non-Hispanic Black, lived in core metropolitan areas, had multiple chronic conditions or outpatient visits in 2010, or saw a nephrologist. In the subset of 18,749 beneficiaries with CKD stage identified from both labs and diagnoses, 70.0% had concordant CKD stage, which was more likely if beneficiaries were older adults, male, lived in micropolitan areas instead of non-core areas, or saw a nephrologist.

**Conclusions:**

There is significant under-diagnosis of CKD in Medicare FFS beneficiaries, which can be addressed with the availability of lab results.

## Background

Chronic kidney disease (CKD) is a costly and increasingly prevalent condition that results in poor health outcomes for many individuals [1]. Documented CKD in Medicare beneficiaries has steadily increased from 2.7% in 2000 to 10.0% in 2011 and 11.0% in 2014, with stage 3 CKD being the fastest growing subgroup [2]. Prior work has shown disparities in CKD prevalence, such that Black or elderly individuals have increased risk for CKD development and progression [2–6].

However, CKD prevalence is likely underestimated [7, 8], especially by stage, with over one-third of beneficiaries with documented CKD lacking specific staging [2]. Moreover, estimates of CKD prevalence are typically based on International Classification of Diseases (ICD)-9 codes, which have low sensitivity and do not identify a majority of individuals with CKD [9–13] compared to laboratory-based measurement of kidney function.

A limited number of studies have assessed patterns of both CKD-related labs and diagnoses. Winkelmayer and colleagues examined lab values and diagnoses of 1852 Medicare beneficiaries hospitalized for myocardial infarction in 1999–2000 in Pennsylvania and found the sensitivity of a range of diagnosis-based identification strategies peaked at 20.7%, while specificity and positive predictive values were consistently above 96% [9]. In a small cohort of patients receiving laboratory testing in the Midwest in 2002–2003, Stevens and colleagues found 11% sensitivity and 96% specificity of diagnostic codes compared to lab-indicated CKD [7]. Ferris and colleagues compared medical chart, laboratory reported, and diagnostic codes of hospitalized patients in a single center and found diagnostic code sensitivity of 50 and 88% specificity [8]. In the cohort of Medicare beneficiaries in the Reasons for Geographic and Racial Differences in Stroke (REGARDS) Study, only 15.5% of patients with lab-identified CKD had Medicare claims with a CKD diagnosis [10]. These studies demonstrate poor identification of CKD from diagnostic coding under specific clinical contexts, but laboratory values have not been comprehensively linked to Medicare claims. Further, limited research has focused on identification of factors independently associated with recognition of CKD or concordance of lab and diagnosis-based CKD identification.

With the availability of 2011 laboratory results linked to Medicare fee-for-service (FFS) claims [11], we can examine the potential value of improving upon population surveillance of CKD in two ways. First, we can identify the extent of clinically unrecognized CKD in a large cohort of Medicare beneficiaries from 10 states in 2011 and patient factors associated with clinical recognition. Given the prior literature noted above, we expected to find significant under-recognition of CKD from claims-based diagnosis codes. Second, we can examine the concordance in CKD staging as reported from lab-based estimated glomerular filtration rates (eGFR) sources and ICD-9 diagnosis codes among those with CKD identified from both sources. We expected to find similar CKD staging from both data sources (i.e. concordance) or a later CKD stage in lab data than diagnosis data. We also examined factors associated with concordance in CKD staging in the two sources.

Results from this analysis provide insights about approaches to CKD recognition, which have implications for the value of comprehensive laboratory data for population surveillance and management. Prior work has demonstrated that CKD is under-recognized in non-Medicare populations, but the extent of under-recognition in Medicare beneficiaries is important to evaluate because 18.5% of Medicare beneficiaries were diagnosed with CKD in 2015 [14]. CKD is costly and puts older adults at risk for ESRD, and Medicare does not obtain laboratory results despite being the largest payer of laboratory testing in the United States. Given significant disparities in the prevalence and management of CKD [4, 15–17], lab-based identification of CKD can improve recognition and may help mitigate known disparities in CKD care. Further, as Centers for Medicare and Medicaid Services (CMS) integrates social determinants of health into value-based purchasing models (including Black race, Hispanic ethnicity, dual eligibility, disability and rural residence) [18], identifying and addressing disparities in these factors is increasingly important.

## Methods

### Data source and cohort

The cohort of Medicare beneficiaries was identified from Medicare FFS claims for beneficiaries in 10 eastern United States (U.S.) states (NY, NJ, MD, DE, VA, NC, SC, GA, FL, AL) [11], which was accessed through the CMS Chronic Conditions Data Warehouse Virtual Research Data Center. The outpatient and carrier files contain institutional and non-institutional provider claims, respectively, for services covered under Medicare Part B, including ICD, Ninth Revision, and Clinical Modification (ICD-9-CM) diagnostic codes and place of service, which were used for the diagnosis-based indication of cohort eligibility. Denominator files provide information on demographic characteristics, birth and death dates, program eligibility and enrollment. Medicare claims for laboratory services were linked to actual outpatient laboratory values processed by a national laboratory vendor in 2011 for Medicare beneficiaries.

**Fig. 1:**
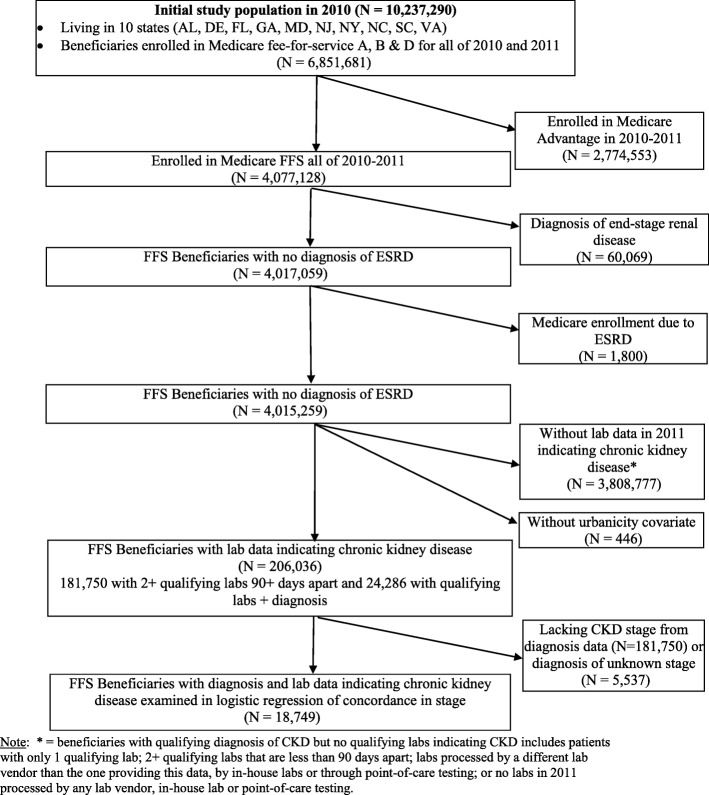
Consort Figure for Medicare FFS Cohort. Note: * = beneficiaries with qualifying diagnosis of CKD but no qualifying labs indicating CKD includes patients with only 1 qualifying lab; 2+ qualifying labs that are less than 90 days apart; labs processed by a different lab vendor than the one providing this data, by in-house labs or through point-of-care testing; or no labs in 2011 processed by any lab vendor, in-house lab or point-of-care testing

There were 10,237,290 Medicare FFS beneficiaries from the 10 states and 6,851,681 were enrolled in Medicare Parts A, B and D between January 1, 2010 and December 30, 2011 (Fig. 1). We excluded beneficiaries if they were enrolled in Medicare Advantage any time in 2010–2011, had no Medicare diagnosis claims during the study period (*n* = 2,774,553), lacked lab-based indicators of CKD (e.g. eGFR < 90 ml/min/1.73m^2^ or albuminuria; *n* = 3,808,777), or were missing the urbanicity covariate (derived from available zip codes; *n* = 446). We also excluded beneficiaries with a diagnosis of ESRD (*n* = 60,069) or who were enrolled in Medicare due to ESRD (*n* = 1800), as dialysis-dependent CKD documentation and care may differ greatly from pre-dialysis CKD. There were 206,036 FFS beneficiaries with CKD in 2011 identified from lab values (Analytic cohort 1). CKD was identified from eGFR values and albuminuria quantifications calculated from the latest serum and urine values in 2011 and classified based on the Kidney Disease Improving Global Outcomes (KDIGO) guidelines (see Table 1, supplemental content, that provides coding rules) [19].

**Table 1:** Descriptive statistics of analytic cohort 1

	Overall Analytic Cohort 1 (*n* = 206,036)	CKD Identified from lab data only (*n* = 181,750)	CKD Identified from lab + Diagnosis (*n* = 24,286)
Stage of CKD identified in lab data *(N*, %)^a^			
Stage 1	612 (0.3%)	549 (0.3%)	63 (0.3%)
Stage 2	115,817 (56.2%)	112,809 (62.1%)	3008 (12.4%)
Stage 3	79,649 (38.7%)	64,460 (35.5%)	15,189 (62.5%)
Stage 4	9152 (4.4%)	3793 (2.1%)	5359 (22.1%)
Stage 5	806 (0.4%)	139 (0.1%)	667 (2.8%)
Age (Mean, SD)	74.3 (9.1%)	74.3 (9.0%)	74.4 (9.5%)
Age Group (*N*, %)			
Age < 65	17,397 (8.4%)	14,920 (8.2%)	2477 (10.2%)
Age 65–69	40,481 (19.7%)	36,332 (20.0%)	4149 (17.1%)
Age 70–74	49,365 (24.0%)	43,996 (24.2%)	5369 (22.1%)
Age 75–79	40,546 (19.7%)	35,619 (19.6%)	4927 (20.3%)
Age 80+	58,247 (28.3%)	50,883 (28.0%)	7364 (30.3%)
Gender (*N*, %)			
Female	129,424 (62.8%)	116,011 (63.8%)	13,413 (55.2%)
Male	76,612 (37.2%)	65,739 (36.2%)	10,873 (44.8%)
Race/Ethnicity (*N*, %)			
Hispanic	7005 (3.4%)	6074 (3.3%)	931 (3.8%)
Non-Hispanic white	170,259 (82.6%)	152,192 (83.7%)	18,067 (74.4%)
Non-Hispanic black	24,153 (11.7%)	19,358 (10.7%)	4795 (19.7%)
Others	4619 (2.2%)	4126 (2.3%)	493 (2.0%)
Original Reason for Medicare Eligibility (*N*, %)			
Age	188,585 (91.5%)	166,783 (91.8%)	21,802 (89.8%)
Disability	17,451 (8.5%)	14,987 (8.2%)	2484 (10.2%)
Medicaid Enrolled (*N*, %)			
No	176,158 (85.5%)	156,172 (85.9%)	19,986 (82.3%)
Yes	29,878 (14.5%)	25,578 (14.1%)	4300 (17.7%)
Geographic Location (*N*, %)			
Large Central Metro	25,785 (12.5%)	22,856 (12.6%)	2929 (12.1%)
Large Fringe Metro	75,775 (36.8%)	66,437 (36.6%)	9338 (38.5%)
Medium Metro	41,155 (20.0%)	36,171 (19.9%)	4984 (20.5%)
Small Metro	21,118 (10.3%)	18,664 (10.3%)	2454 (10.1%)
Micropolitan	20,055 (9.7%)	17,460 (9.6%)	2595 (10.7%)
Non-core area	22,148 (10.8%)	20,162 (11.1%)	1986 (8.2%)
Chronic Conditions in 2010 (*N*, %)			
0–1	11,814 (5.7%)	11,283 (6.2%)	531 (2.2%)
2–3	53,676 (26.1%)	49,853 (27.4%)	3823 (15.7%)
4–5	74,894 (36.4%)	66,607 (36.7%)	8287 (34.1%)
6+	65,652 (31.9%)	54,007 (29.7%)	11,645 (48.0%)
Outpatient Visits in 2010 (*N*, %)			
0–6	63,580 (30.9%)	59,705 (32.9%)	3875 (15.9%)
7–11	57,634 (28.0%)	40,993 (22.6%)	6902 (28.4%)
12–18	47,895 (23.3%)	29,501 (16.2%)	7426 (30.6%)
19+	36,927 (17.9%)	51,551 (28.4%)	6083 (25.1%)
Saw Nephrologist in 2010 (N, %)	18,995 (9.2%)	5980 (3.3%)	13,015 (53.6%)

^a^ CKD Stages: Stage 1 = estimated glomerular filtration rate (eGFR) ≥90 ml/min/1.73m^2^ and urine albumin-to-creatinine ratio (UACR) ≥30 mg/g; Stage 2 = eGFR 60–89; Stage 3 = eGFR 30–59; Stage 4 = eGFR 15–29; Stage 5 = eGFR < 15

The overall cohort of 206,036 beneficiaries included two groups (Fig. 1): 1) beneficiaries who had two or more serum creatinine values at least 90 days apart with eGFR < 90 ml/min/1.73m^2^ based on the CKD-EPI equation [20] or with eGFR ≥90 ml/min/1.73m^2^ and at least one urine albumin value with an albumin-to-creatinine ratio (UACR) ≥ 30 mg/g, but no CKD diagnosis-based indicator of CKD (*n* = 181,750) and, 2) beneficiaries who had both lab-based and diagnosis-based indicators of CKD (*n* = 24,286, Table 2, supplemental content). Note that beneficiaries were determined to have CKD stage 1 if they had an eGFR ≥90 and UACR ≥30, while beneficiaries with eGFR ≥90 and UACR < 30 were excluded from the cohort.

**Table 2:** Model estimated predictors of clinical recognition via diagnosis codes for chronic kidney disease (*n* = 206,036)

	Odds Ratio (95% Confidence Interval)
Stage of CKD identified in lab data (ref: Stage 1)^a^	
Stage 2	0.33 (0.25, 0.44)
Stage 3	2.41 (1.81, 3.20)
Stage 4	10.14 (7.58, 13.57)
Stage 5	23.06 (16.09, 33.05)
Age (ref: < 65)	
Age 65–69	0.80 (0.75, 0.87)
Age 70–74	0.73 (0.68, 0.78)
Age 75–79	0.66 (0.62, 0.71)
Age 80+	0.52 (0.49, 0.56)
Male (ref: Female)	1.62 (1.56, 1.68)
Race/Ethnicity (ref: Non-Hispanic white)	
Hispanic	1.61 (1.46, 1.76)
Non-Hispanic black	1.93 (1.84, 2.03)
Others	0.95 (0.83, 1.07)
Urbanicity (ref: Non-core area)	
Large Central Metro	1.16 (1.07, 1.25)
Large Fringe Metro	1.24 (1.16, 1.33)
Medium Metro	1.21 (1.13, 1.29)
Small Metro	1.27 (1.17, 1.37)
Micropolitan	1.38 (1.28, 1.49)
Chronic Conditions (ref: 0–1)	
2–3	1.24 (1.01, 1.25)
4–5	1.31 (1.18, 1.46)
6+	1.43 (1.29, 1.60)
Medicaid Enrolled (ref: No)	1.10 (1.04, 1.15)
Outpatient Visits in 2010 (ref: 0–6)	
7–11	1.36 (1.29, 1.43)
12–18	1.55 (1.47, 1.64)
19+	1.85 (1.74, 1.96)
Saw Nephrologist in 2010 (ref: No)	16.65 (15.99, 17.34)

^a^ CKD Stages: Stage 1 = estimated glomerular filtration rate (eGFR) ≥90 ml/min/1.73m^2^ and urine albumin-to-creatinine ratio (UACR) ≥30 mg/g; Stage 2 = eGFR 60–89; Stage 3 = eGFR 30–59; Stage 4 = eGFR 15–29; Stage 5 = eGFR < 15

### Covariates

Patient factors in this analysis included lab-identified CKD stage 1–5, age group (< 65, 65–69, 70–74, 75–79, 80+), sex, race/ethnicity (non-Hispanic White, Hispanic, non-Hispanic Black, Other), Medicaid dual enrollment, number of chronic conditions, and urbanicity. Multiple chronic conditions (0–1, 2–3, 4–5, 6+) were constructed from the CCW chronic disease flags at end-of-year for 18 conditions in 2010 [21]. We used the National Center for Health Statistics’ (NCHS) 6-level urban-rural classification (large central metro, large fringe metro, medium metro, small metro, micropolitan, non-core) to define urbanicity according to county of residence for Medicare beneficiaries. From carrier physician claims, we calculated the number of outpatient visits for Evaluation and Management (E&M) visits in 2010 based on Healthcare Common Procedure Coding System (HCPCS) codes 992.xx, 993.xx and 994.xx. Finally, we determined whether each beneficiary was seen by a nephrologist during an outpatient visit in 2010 as these providers may be particularly attuned to recognizing CKD.

### Outcomes

The first outcome of interest was clinical recognition of CKD defined based on the presence of diagnosis codes, which was constructed on the cohort of 206,036 FFS beneficiaries with lab-identified CKD (Analytic cohort 1). A beneficiary was indicated to have clinically recognized CKD (i.e. the provider was aware of CKD and submitted a claim for it) if there was at least one diagnosis code from 2011 Medicare claims indicating CKD stages 1–5 (ICD-9 codes 585.1–585.5) or unknown stage (ICD-9 codes 250.4x, 403.9x or 585.9).

The second outcome of interest was a binary indicator of concordance in CKD staging between lab and diagnosis data. Of the 24,286 beneficiaries with both lab-based and diagnosis-based indicators of CKD, 5537 beneficiaries were excluded due to diagnosis of unknown stage (ICD-9 codes 250.4x, 403.9x or 585.9), leaving a subsample of 18,749 beneficiaries with both lab-based and diagnosis-based indicators for CKD from which stage could be identified (Analytic cohort 2).

### Statistical analysis

Study cohort characteristics were summarized using mean and standard deviation for continuous variables and frequency and percent for categorical. To examine patient factors associated with clinically recognized CKD via diagnoses in the overall cohort (*n* = 206,036), we estimated a logistic regression using several patient factors hypothesized to be related to clinical CKD recognition. To examine patient factors associated with concordance, we included all covariates listed above except for CKD stage. Three logistic regression models were performed using SAS version 9.4 LOGISTIC procedure (SAS Institute Inc., Cary, North Carolina) using sociodemographic factors (Model 1), coupled with comorbidity (Model 2) and healthcare access and utilization factors (Model 3), where each model included the covariates in the prior model, to better isolate the effects of each factor type. Statistical significance was determined using a two-sided 0.05 significance level and all analyses were specified a priori. The institutional review board of Duke University approved the study.

## Results

### Clinical recognition of CKD (analytic cohort 1)

In the overall cohort of 206,036 beneficiaries (Analytic cohort 1), the mean age in all groups was 74 (Table 1). Only 11.8% (*n* = 24,286) had clinically recognized CKD via diagnoses. Of those with clinically recognized CKD 12.7% had stage 1 or 2 CKD (versus 62.4% in the lab-only identified CKD cohort), 10.2% were non-elderly (vs. 8.2%), 55.2% were female (vs. 63.8%), and 74.4% were non-Hispanic white (vs. 83.7%). The majority (89.8%) were age eligible for Medicare (vs. 91.8%), 17.7% were dually enrolled in Medicaid (vs. 14.1%), 8.2% resided in non-core (e.g., rural) areas (vs. 11.1%), and 48.0% had 6+ conditions (vs. 29.7%). Over half (53.6%) of clinically recognized beneficiaries had seen a nephrologist, compared to only 3.3% of lab only-identified beneficiaries.

In logistic regression (Table 2), beneficiaries were more likely to have clinically recognized CKD if they had higher CKD stages (stage 3: odds ratio (OR) = 2.41, 95% confidence interval (CI): 1.81–3.20; stage 4: OR = 10.14, 95% CI: 7.58–13.57; stage 5: OR = 23.06, 95% CI: 16.09–33.05, compared to stage 1) or saw a nephrologist (OR = 16.65, 95% CI: 15.99–17.34). Beneficiaries were also more likely to have clinically recognized CKD if they were Hispanic (OR = 1.61, 95% CI: 1.46–1.76), Non-Hispanic Black (OR = 1.93, 95% CI: 1.84–2.03), dually enrolled in Medicaid (OR = 1.10, 95% CI: 1.04–1.15), had multiple chronic conditions (2–3 conditions: odds ratio (OR) = 1.24, 95% confidence interval (CI): 1.01–1.25; 4–5 conditions: OR = 1.31, 95% CI: 1.18–1.46; 6+ conditions: OR = 1.43, 95% CI: 1.29–1.60, compared to 0–1 conditions), had many outpatient visits in 2010 (7–11 visits: OR = 1.36, 95% CI: 1.29–1.43; 12–18 visits: OR = 1.55, 95% CI: 1.47–1.64; 19+ visits: OR = 1.85, 95% CI: 1.74–1.96, compared to 0–6 visits) or lived in core areas. Beneficiaries were less likely to have their CKD clinically recognized if they were older adults (65–69 years: odds ratio (OR) = 0.80, 95% confidence interval (CI): 0.75–0.87; 70–74 years: OR = 0.73, 95% CI: 0.68–0.78; 75–79 years: OR = 0.66, 95% CI: 0.62–0.71; 80+ years: OR = 0.52, 95% CI: 0.49–0.56, compared to < 65 years).

### Concordance between lab-based and diagnosis-based definitions of CKD (analytic cohort 2)

In the subset of 18,749 beneficiaries with CKD of known stage identified in (Analytic cohort 2), the most prevalent group were beneficiaries with stage 3 CKD regardless of identification by labs or diagnoses, followed by stages 4 and 2, respectively (Table 3). As expected, there was significant concordance between lab-based and diagnosis-based staging. Across all stages, 70.0% of this subset had concordant CKD stage by lab and diagnosis data. More beneficiaries had a higher CKD stage from lab data than from diagnosis data (19.4%) and fewer beneficiaries had a higher stage from diagnoses than labs (10.6%).

**Table 3:** Analytic cohort 2 concordance in chronic kidney disease staging between lab data and diagnoses from claims (*n* = 18,749)

Lab-based Stages ↓	ICD-9-based Stages				
	Stage 1	Stage 2	Stage 3	Stage 4	Stage 5
Stage 1 confirmed, eGFR> = 90 and UACR> = 30	8	**7** ^a^	**5** ^a^	**0** ^a^	**0** ^a^
Stage 2	**177** ^b^	673	**1017** ^a^	**39** ^a^	**5** ^a^
Stage 3	**232** ^b^	**994** ^b^	9730	**791** ^a^	**38** ^a^
Stage 4	**34** ^b^	**69** ^b^	**1785** ^b^	2475	**89** ^a^
Stage 5	**5** ^b^	**5** ^b^	**51** ^b^	**286** ^b^	234

Unbolded cells indicate lab-based CKD stage = ICD-9-based CKD stage (concordance, *n* = 13,120 [70.0%]); bolded cells with ^a^ indicate lab-based CKD stage < ICD-9-based CKD stage (discordance, *n* = 1991 [10.6%]); bolded cells with ^b^ indicate lab-based CKD stage > ICD-9-based CKD stage (discordance, *n* = 3638 [19.4%])

In logistic regression of CKD staging concordance (Table 4), beneficiaries in the lab + diagnosis cohort were more likely to have their CKD staged identically in labs and diagnoses if they were older adults (age 65–69: odds ratio (OR) = 1.33, 95% confidence interval (CI): 1.17–1.51; age 70–74: OR = 1.28, 95% CI: 1.14–1.45; age 75–79: OR = 1.36, 95% CI: 1.20–1.53; age 80+: OR = 1.20, 95% CI: 1.07–1.35, compared to age < 65), male (OR = 1.17, 95% CI: 1.10–1.25), lived in micropolitan areas compared to non-core areas (OR = 1.38, 95% CI: 1.20–1.60) or saw a nephrologist in 2010 (OR = 1.25, 95% CI: 1.17–1.34). There were no concordance disparities by race/ethnicity, multi-morbidity or prior outpatient utilization. These results were consistent across changes in covariate specification.

**Table 4:** Analytic cohort 2 logistic regression of concordance between lab-based and diagnosis-based CKD stage (*n* = 18,749)

	Odds Ratio (95% Confidence Interval)		
	Model 1	Model 2	Model 3
Age < 65 (ref)	–	–	–
Age 65–69	1.35 (1.19, 1.52)	1.34 (1.19, 1.52)	1.33 (1.17, 1.51)
Age 70–74	1.30 (1.15, 1.46)	1.30 (1.15, 1.46)	1.28 (1.14, 1.45)
Age 75–79	1.36 (1.21, 1.54)	1.37 (1.21, 1.54)	1.36 (1.20, 1.53)
Age 80+	1.20 (1.07, 1.35)	1.21 (1.08, 1.35)	1.20 (1.07, 1.35)
Male (ref: Female)	1.18 (1.11, 1.26)	1.18 (1.11, 1.26)	1.17 (1.10, 1.25)
Race/Ethnicity (ref: Non-Hispanic white)			
Hispanic	1.09 (0.91, 1.31)	1.09 (0.91, 1.31)	1.13 (0.94, 1.11)
Non-Hispanic black	1.03 (0.95, 1.12)	1.03 (0.95, 1.11)	1.02 (0.94, 1.11)
Others	1.07 (0.85, 1.35)	1.07 (0.85, 1.35)	1.08 (0.86, 1.36)
Urbanicity (ref: Non-core area)			
Large Central Metro	1.10 (0.95, 1.28)	1.11 (0.96, 1.28)	1.10 (0.95, 1.27)
Large Fringe Metro	1.13 (1.00, 1.28)	1.14 (1.01, 1.28)	1.10 (0.98, 1.25)
Medium Metro	1.01 (0.88, 1.14)	1.01 (0.89, 1.15)	0.98 (0.86, 1.12)
Small Metro	1.10 (0.95, 1.27)	1.10 (0.95, 1.27)	1.08 (0.93, 1.26)
Micropolitan	1.41 (1.21, 1.63)	1.41 (1.22, 1.63)	1.38 (1.20, 1.60)
Chronic Conditions (ref: 0–1)			
2–3	–	1.00 (0.80, 1.24)	0.95 (0.76, 1.19)
4–5	–	1.08 (0.87, 1.34)	1.01 (0.81, 1.26)
6+	–	1.00 (0.80, 1.23)	0.93 (0.72, 1.16)
Medicaid Enrolled (ref: No)	–	–	0.96 (0.87, 1.05)
Outpatient Visits in 2010 (ref: 0–6)			
7–11	–	–	1.03 (0.92, 1.14)
12–18	–	–	0.98 (0.87, 1.10)
19+	–	–	1.01 (0.91, 1.12)
Saw Nephrologist in 2010 (ref: No)	–	–	1.25 (1.17, 1.34)

## Discussion

This is the first study to examine the extent to which CKD is clinically unrecognized via diagnoses in a cohort of Medicare FFS beneficiaries with lab-identified CKD by examining concordance between CKD stages reported from lab values and ICD-9 diagnoses in Medicare FFS claims. Such a comparison has not been possible heretofore because lab results had never been linked to FFS claims. This analysis found that only 11.8% of beneficiaries with lab-identified CKD had a corresponding diagnosis despite having lab values indicating CKD, which is consistent with prior work in non-Medicare populations that CKD is under-diagnosed [22, 23]. While a majority of beneficiaries with clinically unrecognized CKD had earlier stages of disease, there was a large percentage (37.8%) with unrecognized moderate to severe CKD (stages 3–5) - levels of kidney function for which intervention could slow progression or enable timely preparation for renal replacement therapy.

Despite epidemiologic evidence of worsened outcomes among racial/ethnic minorities with CKD compared to white counterparts [5, 24], our study found CKD recognition via diagnostic codes to be higher among Hispanic and non-Hispanic black beneficiaries than non-Hispanic whites. Others have similarly noted recognition of CKD to be higher among minorities, possibly due to providers’ heightened recognition of risk among individuals with higher prevalence of CKD risk factors (e.g., diabetes and hypertension) [25]. However, recognition alone, without concomitant adherence to CKD guideline recommendations, has not improved clinical outcomes among high risk groups [25, 26]. Further, our study found CKD recognition to be lower among elderly beneficiaries compared to non-elderly beneficiaries. While distinguishing between true kidney dysfunction and age-related renal decline remains challenging, overall CKD prevalence rises in parallel with age [27]. Therefore, as life expectancies continues to lengthen, efforts must be directed toward attenuating the sustained increased risk of adverse clinical outcomes, such as cardiovascular disease, progression to end-stage renal disease (ESRD), and death in the growing elderly population with CKD compared to those without CKD [2, 28].

Several factors may partly explain suboptimal provider recognition of CKD, including lack of provider understanding of CKD and CKD risk categories, and poor knowledge of appropriate CKD management [29, 30]. Despite availability of practice guidelines, providers are unfamiliar with them or prioritize management of co-morbid conditions over that of CKD [30–32]. Although the automated reporting of eGFR modestly increased overall CKD recognition, recognition among high risk Black or older patients remained unaffected [33]. Our study found that being seen by a nephrologist in the prior year (2010) was strongly associated with having CKD clinically recognized in 2011, which is consistent with prior studies that found higher rates of appropriate testing, medication management, and preparation for renal replacement therapy among individuals engaged in nephrology care (versus not-engaged) [34–36]. It is also possible that patients had competing clinical demands, limiting time and sufficient attention with providers to enable CKD diagnosis. Early identification and management of CKD is paramount to improving outcomes, particularly among those at highest risk of progression, and provides an opportunity for earlier patient education, avoidance of nephrotoxins and acute kidney injury, implementation of behavioral and medication management strategies to slow progression, and referral to specialist care [37–39].

In the analysis of concordance between lab-based and diagnosis-based indicators of CKD, we found that most beneficiaries had similar CKD staging. We also found that older or male beneficiaries were significantly more likely to have indicator concordance than those < 65 or females, respectively. This latter finding is of particular interest because nationally, females constitute the majority of Medicare FFS beneficiaries [40] and have higher national prevalence estimates of pre-dialysis CKD compared to males. However, recognition and appropriate documentation of CKD in females is worse than in males [41, 42], suggesting a gender disparity in CKD care. Interestingly, there was no relation between race/ethnicity and concordance after adjustment, highlighting the potential for lab-based identification of disease to attenuate, to some extent, social influences historically associated with provider-driven disparities in diagnosis-based recognition of disease [43]. Nephrologists were also a significant predictor of concordance, such that beneficiaries seeing a nephrologist in 2010 had 25% higher odds of concordance in 2011 than those without a nephrologist visit.

This work demonstrates that laboratory data offer three distinct opportunities for identifying CKD on a population basis. First, serum creatinine values converted to eGFR values can identify CKD that is clinically unrecognized because it is not diagnosed. In population surveillance efforts, the addition of laboratory data will enable appropriate recognition of CKD. Further, lab-based identification can avoid under-treatment of beneficiaries with advanced CKD who are at risk for adverse health events and significant health care utilization. Second, lab-based identification of CKD can more accurately indicate CKD stage when providers use the diagnosis codes for CKD of unknown stage. This was less common than clinically unrecognized CKD, but is still important for identifying patients in need of closer management. Third, lab-based CKD stage was concordant with diagnosis-based CKD in the vast majority of patients identified in both data sources. When there was discordance, lab data often indicated a higher stage of CKD than diagnoses from Medicare claims. This may reflect providers not updating billing documentation, despite actually observing disease progression and managing risk, or initial CKD identification by providers without subsequent recognition of CKD progression.

There are several limitations that must be acknowledged. We cannot say with absolute certainty that lack of a diagnosis code indicates unrecognized CKD; it is feasible that there is clinical recognition that does not rise to the level of a formal diagnosis code but is notated elsewhere on a patient’s medical record problem list. In fact, receipt of a nephrology consultation is likely evidence of CKD recognition, although this cannot be truly confirmed without knowledge of the indication for referral. These results strongly suggest that use of ICD-9 based diagnosis for identifying patients with CKD is questionable to the point of being invalid. Further, our results may have limited generalizability to Medicare FFS beneficiaries outside of these 10 states examined or to years subsequent to 2011. The ability to examine disparities in CKD staging was limited because the lab results data was available from only one national laboratory vendor, albeit the vendor with the largest market share in these 10 states [11]. However, it should be noted that the sample size in the concordance analysis exceeds the prior two studies [9, 10] by a factor of 100. These results do not reflect all laboratory data however this single laboratory vendor’s market share for Medicare laboratory tests in these 10 states in 2011 ranged from 9 to 51%. However, data capture would not converge to 100% because of in-house laboratory and point-of-care laboratory testing reimbursed by Medicare. Serum creatinine values are only available in this sample if a physician ordered the test, a Medicare FFS beneficiary followed through and had the test done, and the test was processed by a clinical facility affiliated with this single lab vendor [44]. Similarly, the use of just one qualifying UACR measurement may misclassify individuals as having CKD Stage 1 instead of a transient urinary alteration, as can be seen with a urinary infection or acute kidney injury, rather than persistent albuminuria (> 3 months) consistent with CKD. Further, our list of included Medicare claims covariates likely do not capture all factors that may explain variation in CKD diagnosis, such as medication use patterns or iatrogenic factors. Last, staging was based on most recent creatinine values which may represent transient fluctuations in kidney function from superimposed acute kidney injury on CKD rather than stable CKD.

## Conclusions

These results have research and policy implications. Possible linkage of longitudinal lab data to Medicare claims would allow exploration of the continuum of CKD progression and identify targets for early interventions for CKD disease management. As the single largest payer of laboratory tests in the United States [45], linkage of Medicare with lab data facilitates population surveillance. Further, investment in preventive strategies earlier in CKD care may offset the substantial downstream costs by slowing or preventing progression to ESRD, or by prioritization of management strategies and resources for individuals at greatest risk of adverse outcomes based on more than eGFR alone [46]. These results indicate that the availability of lab data has tremendous value for population surveillance of CKD by significantly reducing the under-recognition of CKD from claims-based diagnoses that are readily available but insufficient to the task.

## Data Availability

The data presented in this manuscript is under the possession of the Centers for Medicare and Medicaid Services (CMS) and cannot be publicly shared.

## Abbreviations

AL: Alabama
CI: confidence interval
CKD: Chronic kidney disease
CKD-EPI: *Chronic Kidney Disease* Epidemiology Collaboration
CMS: Centers for Medicare and Medicaid Services
DE: Delaware
E&M: Evaluation and Management
eGFR: *estimated glomerular filtration rate*
ESRD: End-stage renal disease
FFS: Fee-for-service
FL: Florida
GA: Georgia
HCPCS: Healthcare Common Procedure Coding System
ICD: International Classification of Diseases
KDIGO: Kidney Disease Improving Global Outcomes
MD: Maryland
NC: North Carolina
NJ: New Jersey
NY: New York
OR: odds ratio
REGARDS: Reasons for Geographic and Racial Differences in Stroke
SC: South Carolina
VA: Virginia
